# Splenic Metastasis of Squamous Cell Carcinoma of the Uterine Cervix: A Case Report and Review of the Literature

**DOI:** 10.1155/2014/798948

**Published:** 2014-07-24

**Authors:** Shigeki Taga, Mari Sawada, Aya Nagai, Dan Yamamoto, Ryoji Hayase

**Affiliations:** Department of Obstetrics and Gynecology, National Hospital Organization Fukuyama Medical Center, Okinogamicho 4-14-17, Fukuyama, Hiroshima Prefecture 0825-720, Japan

## Abstract

Metastasis from various neoplasms to the spleen is very rare and most of the cases are found at autopsy. We report a patient presenting with uterine cervical cancer with splenic metastases. A 49-year-old woman presenting with genital bleeding was referred to our hospital and diagnosed with stage IIB cervical cancer. She underwent concomitant chemoradiotherapy (CCRT) consisting of 50 Gy whole pelvis irradiation, high-dose-rate intracavitary brachytherapy 24 Gy/4 fractions and six weeks of paclitaxel and carboplatin administration. Ten months after the initial therapy, CT revealed recurrence at spleen. Although she received 5 courses of nedaplatin, enlargement of the tumor was noticed. She underwent a splenectomy and the result of histology was compatible with metastasis of cervical cancer.

## 1. Introduction

Improvement of chemotherapy, irradiation, and surgical treatment has resulted in better control of disease and longer survival of cancer patients and also has led to high incidences of distant metastases. However, metastasis from cervical cancer to the spleen is very rare and most of the cases are found at autopsy. Here we report a case of cervical cancer metastasizing to the spleen.

## 2. Case Report

**Figure 1:**
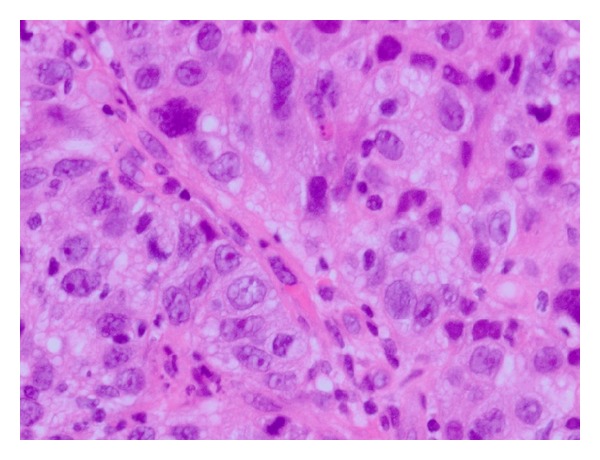
The pathological examination of the biopsy showed undifferentiated carcinoma (H&E ×400).

A 49-year-old woman presented to our department with massive genital bleeding. Vaginal examination revealed a protruding mass in the cervical os. A cervical biopsy was performed and the pathological examination of the biopsy showed undifferentiated carcinoma (Figure 1).

**Figure 2:**
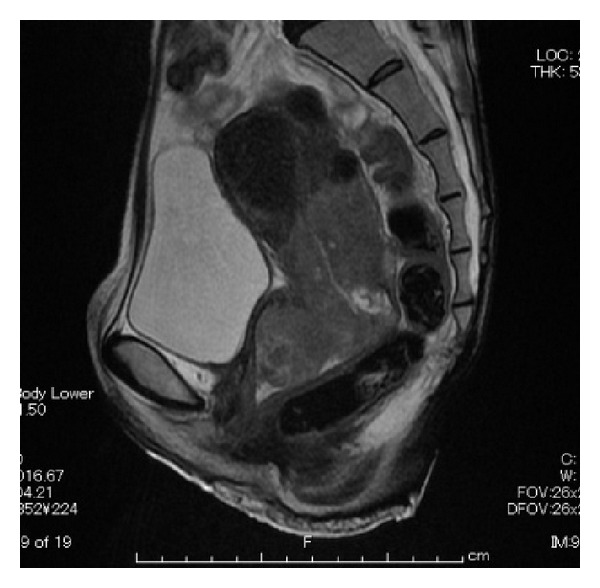
MRI (T2-weighted) reveals a large mass infiltrating to the uterine corpus and vagina.

She was diagnosed with stage IIB cervical cancer by rectal examination and was admitted to our hospital. The levels of squamous cell carcinoma antigen (SCC) and carcinoembryonic antigen (CEA) were elevated to 7.2 ng/mL and 7.18 ng/mL, respectively. Magnetic resonance imaging (MRI) of the pelvis revealed a large mass infiltrating to the uterine corpus and vagina (Figure 2).

**Figure 3:**
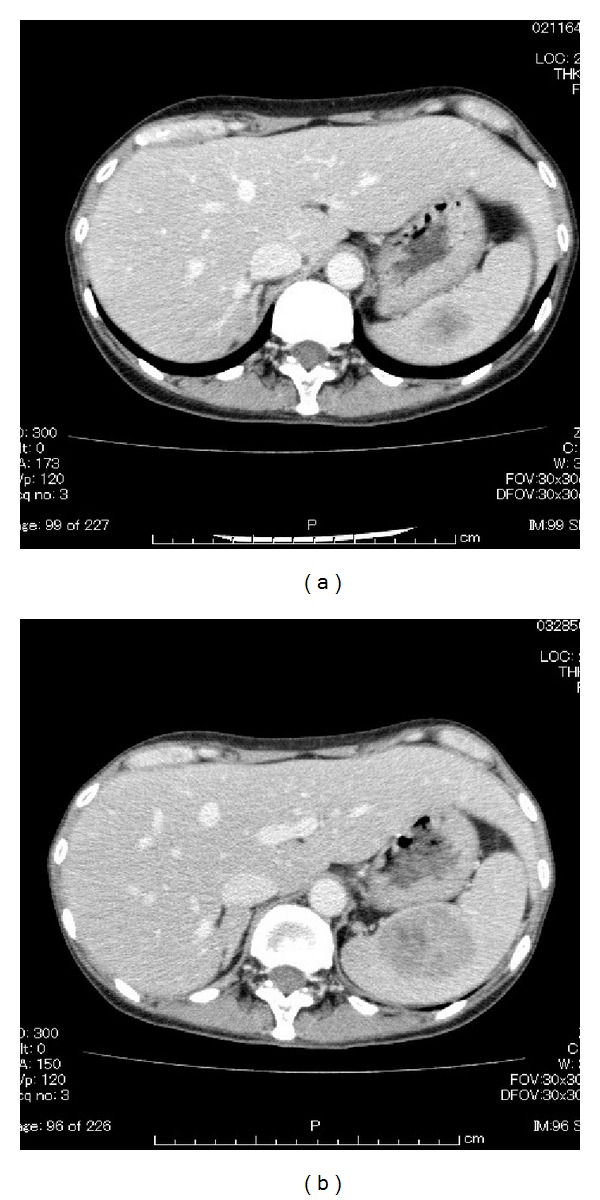
(a) CT showing probable spleen metastasis. (b) The mass found in the spleen enlarged.

Hemoglobin level was 5.0 g/dL and a total of 8 units of packed red blood cells were transfused. She underwent concomitant chemoradiotherapy (CCRT) consisting of 50 Gray whole pelvis irradiation in 25 fractions at 2 Gray per fraction, high-dose-rate intracavitary brachytherapy 24 Gray/4 fractions, and six weeks of paclitaxel (60 mg/m^2^/week) and carboplatin (AUC = 2/week) administration. When the therapy was completed, MRI revealed almost complete remission. The levels of SCC and CEA decreased to normal ranges. Cytological test of the cervix was negative. The patient remained free of disease for 10 months. Then a routine follow-up CT suspected spleen metastasis. Although five courses of nedaplatin (70 mg/m^2^/4 weeks) were administered, the level of SCC was elevated to 19.45 ng/dL and the mass in the spleen enlarged (Figure 3).

**Figure 4:**
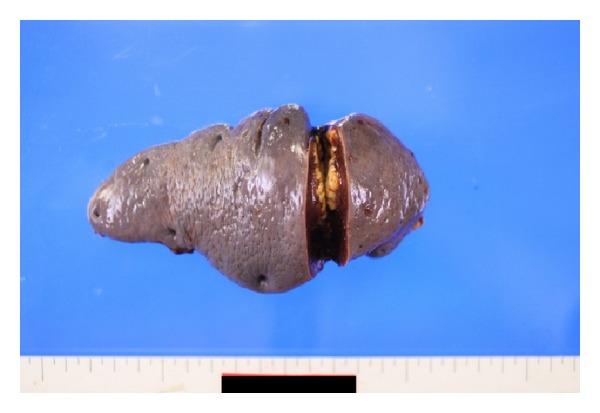
The surgical specimen of splenectomy.

**Figure 5:**
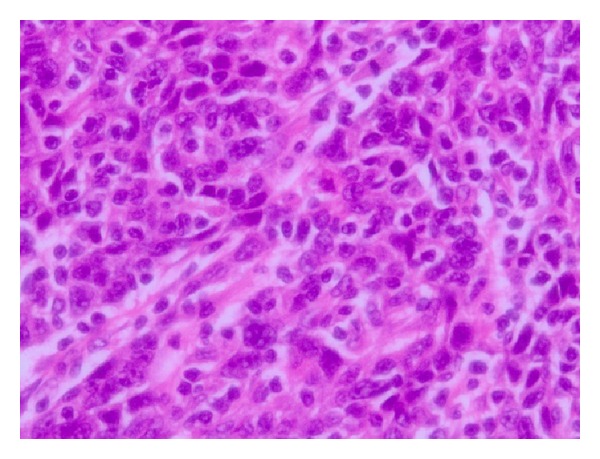
The pathological examination revealed undifferentiated carcinoma identical to the former specimen (H&E ×400).

An exploratory laparotomy was performed and the spleen was removed (Figure 4). The result of pathological examination was compatible with metastasis from cervical cancer (Figure 5).

Four months after the surgery, CT revealed clavicular, axillary, mediastinal, and para-aortic lymph nodes metastases. Three courses of irinotecan (60 mg/m^2^/3 weeks) were administered. Then CT revealed stable disease. She underwent 7 courses of systemic chemotherapy consisting of irinotecan (60 mg/m^2^/3 weeks) and nedaplatin (70 mg/m^2^/3 weeks). CT revealed stable disease again. Five courses of paclitaxel and carboplatin were administered. She is alive with the disease 20 months after splenectomy.

## 3. Discussion

Carlson et al. reported in their review that overall incidence of distant metastasis of 2200 cases of squamous cell carcinoma of the cervix cancer was 15.3% [1]. Cervical cancer usually spreads by local invasion or via lymphatics to the pelvic lymph nodes. Hematogenous metastasis is rather rare. The most commonly reported sites of distant metastasis are the lung, bone, mediastinal and supraclavicular nodes, and liver [1, 2].

Metastasis from cervical cancer to the spleen is very rare and most of the cases are found at autopsy. Spleen metastasis at autopsy is reported to be 1.6% to 30% [3]. When the spleen metastasis is found, there are usually multiple organ metastases and surgery is not indicated. Uterine cervical cancer is a rare source of spleen metastasis [4]. The first case was reported by Brufman et al. in 1977 [5]. The second case was by Klein et al. [6]. In many cases, cervical cancer metastasizes to the pelvic and para-aortic lymph nodes before remote metastases. As this clinical entity is rare, no large clinical data has been reported. Sotto et al. found 8 cases of spleen metastasis in 108 autopsy cases [7]. Badib et al. reported 12 spleen metastases and 16 adrenal metastases in 278 autopsy cases of cervical cancer [8]. Lam et al. reported 0.63% of spleen metastasis in 92 autopsy cases of various cancer [9].

Goktolga et al. reported that the interval between initial treatment and spleen metastasis was 20–42 months [3]. Klein et al. reported 20–24 months [6]. As for clinical symptoms, Carvalho et al. reported a patient who complained of a left hypochondriac pain radiating to the ipsilateral shoulder [2]. Lam and Tang reported that only 7 in 92 patients of metastatic splenic tumors had symptoms but two of them had spleen rupture [9].

The present case was followed up with vaginal examination, cytological test, and serum tumor markers every time and CT. The serum levels of SCC and CEA were elevated. SCC is a glycoprotein with a molecular weight of approximately 45 kDa which was first reported by Kato and Torigoe [10]. Pang reported a case of spleen metastasis of cervical cancer suspected on the base of elevated SCC level [11]. Crombach et al. showed that SCC is influenced by the infiltrative growth of the tumor [12]. CEA had been reported by Ikeda et al. to be useful for predicting the status of postsurgical high-risk factors in women with SCC of the uterine cervix [13].

Splenic metastasis of uterine cervical cancer is rare but has poor prognosis. Surgical treatment is indicated in case the patient has symptoms. Many patients have no symptoms but some patients have spleen rupture. All abdominal organs, not only in the pelvic space, must be evaluated for metastasis.
